# Predicting neurodevelopmental outcomes for at-risk infants: reliability and predictive validity using a Chinese version of the INFANIB at 3, 7 and 10 months

**DOI:** 10.1186/1471-2431-12-72

**Published:** 2012-06-14

**Authors:** Wei Liao, En-yi Wen, Chao Li, Qing Chang, Kui-lin Lv, Wang Yang, Zhou-mei He, Cong-min Zhao

**Affiliations:** 1 Department of Pediatrics, Xinqiao Hospital, the Third Military Medical University, Chongqing 400037, China

**Keywords:** Infant, Neurological development, Prediction, Neuromotor abnormality, Chinese population

## Abstract

**Background:**

Chinese primary care settings have a heavy patient load, shortage of physicians, limited medical resources and low medical literacy, making it difficult to screen for developmental disorders in infants. The Infant Neurological International Battery (INFANIB) for the assessment of neuromotor developmental disorders in infants aged 0 ~ 18 months is widely applied in community health service centers because of its simplicity, time-saving advantages and short learning curve. We aimed to develop and assess a Chinese version of the INFANIB.

**Methods:**

A Chinese version of the INFANIB was developed. Fifty-five preterm and 49 full-term infants with high risk of neurodevelopmental delays were assessed using the Chinese version of the INFANIB at 3, 7 and 10 months after birth. The Peabody Developmental Motor Scale (PDMS) was simultaneously used to assess the children with abnormalities and diagnose cerebral palsy. The sensitivity, specificity, positive predictive value and negative predictive value of the scale were calculated.

**Results:**

At birth, a higher proportion of full-term infants had asphyxia (*p* < 0.001), brain damage ( *p* = 0.003) and hyperbilirubinemia ( *p* = 0.022). The interclass correlation coefficient and intraclass correlation coefficient values for the INFANIB at 3, 7 and 10 months were >0.8, indicating excellent reliability with regard to inter- and intraobserver differences. The specificity, sensitivity, positive predictive value and negative predictive value were high for both high-risk premature infants and full-term infants at the age of 10 months. For premature infants at the age of 7 months or below, INFANIB had low validity for detecting abnormalities.

**Conclusions:**

The Chinese version of the INFANIB can be useful for screening infants with high-risk for neuromotor abnormality in Chinese primary care settings.

## Background

The survival of high-risk infants with premature delivery, low birth weight, intrauterine growth retardation, birth asphyxia, intraventricular hemorrhage or chronic lung diseases has increased significantly through the development of medical technology for perinatal care. In 2010, the World Health Organization reported that the incidence of premature delivery is 10%, and 54% of premature infants are born in Asia [1]. Premature infants comprise a special group of high-risk infants. Their motor development is different from that of healthy full-term infants and their incidence of motor disorders is higher than their incidence of recognition and behavioral disorders [2]. Evidence is growing that the first year of an infant’s life is a critical period for brain development due to brain plasticity [3-6]. Motor development during the first year of life is rapid and extensive and is influenced by biological, environmental, and social factors. Interventions during the first year of life can significantly improve the outcome of neuromotor abnormalities.

China has an exceptionally large population, and the incidence of neuromotor abnormalities and cognitive and behavioral disorders in infants is increasing. In China, the community healthcare setting plays a critical role in early identification of neuromotor abnormalities in infants. However, knowledge of medical professionals about neuromotor abnormalities in high-risk infants remains insufficient. Thus, it is imperative to develop a scale that is simple to apply, requires only a short time for assessment and provides favorable reliability and validity for identifying neuromotor abnormalities in infants in Chinese primary care settings.

Currently in China, several scales are applied in limited general hospitals for measuring infant neuromotor development, including the Bayley Scales of Infant Development (BSID-II) [7], the Gesell development schedules [8], and the Peabody Developmental Motor Scales, Second Edition, (PDMS-2) [9]. The Alberta Infant Motor Scale (AIMS) [10] has been investigated but is not yet widely applied in Chinese clinical settings. Other scales such as the Test of Infant Motor Performance (TIMP) [11] and Toddler and Infant Motor Evaluation (TIME) [12] have not yet been introduced in China. Reports of the above mentioned instruments suggest that they are time consuming and require specialized training, which makes them impractical for routine use in China where most communities have an abundance of patients, a shortage of physicians, limited medical resources and low medical literacy. Another investigative tool has been introduced, however, that appears to have wide application in community health service centers because of its simplicity and time-saving advantages, and its short learning curve for physicians, nurses and physical therapists. Ellison and colleagues developed the Infant Neurological International Battery (INFANIB) for the assessment of neuromotor developmental disorders in infants aged 0 ~ 18 months [13]. The original INFANIB was a 20-item battery with five factors: Spasticity, Vestibular Function, Head and Trunk, French Angles and Legs. The INFANIB was validated in Iran and its sensitivity was 90%, specificity 83%, positive predictive value (PPV) 79% and negative predictive value (NPV) 93% [14]. The intraclass correlation coefficient (ICC) was 0.90. Studies have confirmed that the INFANIB is easy to apply and time-saving. North American and Iranian studies have shown that this scale has favorable reliability and validity in the prediction of gross motor development, and its assessment procedures can be mastered by medical personnel of different professions, including clinicians and nurses [14,15].

Differences in attaining gross motor milestones have been noted among different ethnic groups; in one study, Indian Black Caribbean, and Black African children were found less likely to be delayed (in adjusted models), while increased likelihood of delays was seen among Pakistani and Bangladeshi infants, after considering socio-economic factors [16]. Racial and ethnic variations were also noted among American children; black infants were advanced in motor development in their first two years and as they became school age, especially boys, they consistently performed better than white and Hispanic children in activities such as running and vertical jumps, while white and Hispanic children had less consistent performance overall in motor development skills [17]. Considering ethnic variations, we feel that it is appropriate and necessary for the reliability and validity of a Chinese version of the INFANIB to be confirmed in a Chinese population. The present study was carried out to develop a Chinese version of the INFANIB to assess its reliability and validity and to explore its feasibility for screening neuromotor developmental disorders in Chinese primary care settings.

## Methods

### Subjects

A total of 118 infants evaluated at an out-patient clinic and hospitalized for high-risk neurodevelopmental delays from January 2008 to December 2010 were randomly selected for this study. All infants had no genetic abnormalities and had been hospitalized in the neonatal intensive care unit (NICU). These infants were Han Chinese from the Chongqing region of China. The ratio of suburban to urban infants was 4:1. The age at first hospital visit was 1–3 months (corrected age for premature infants). Infants who did not complete follow-up were excluded from the study. One hundred four infants were assessed for neuromotor development at 3, 7 and 10 months and then followed up until they reached age one year. After one year (12–24 months), the PDMS-2 was administered by professional evaluators to assess motor development outcomes and cerebral palsy was diagnosed simultaneously by pediatricians based on severe abnormal motor development (See details below.). If diagnosis of CP is not confirmed, follow up is needed, including repeat evaluation by PDMS-2. For the purpose of analyzing predictive validity, the infants were divided into premature infants (n = 55) and full-term high-risk infants (n = 49).

### Ethical considerations

In compliance with the Helsinki Declaration, the study protocol was reviewed and approved by the Institutional Review Board of the 2^nd^ Affiliated Hospital of the Third Military Medical University, China (No. 2007082) on December 6, 2007. Signed informed consent was provided by the parents or legal guardians of all subjects.

### Definitions

For the purposes of this study, developmental and clinical terms were defined as follows:

*Asphyxia* was defined as infants with fetal acidosis (pH <7.0), a 5-min Apgar score of 0–3. HIE (e.g., altered tone, depressed level of consciousness, seizures) and other multiorgan system signs (e.g., altered consciousness, muscle tone, posture, tendon reflexes/clonust, myclonus present/absent, pupils, seizures, etc.).

*Brain damage* was defined as presence of HIE of full-term infants and periventricular leukomalacia (PVL) or preterm infants.

*Hypoxic ischemic encephalopathy (HIE)* is an important cause of permanent damage to central nervous system cells it may result in neonatal death or manifest as cerebral palsy or mental deficiency. HIE was diagnosed based on: a)profound metabolic or mixed acidemia (pH <7.0) in an umbilical arterial blood sample. b)an Apgar score of 0 to 3 for > 5 min. c) neurological manifestation such as seizure, coma or hypotonia and d) evidence of multiorgan dysfunction.

*Intracranial hemorrhage* may results from trauma or asphyxia and rarely from a primary hemorrhagic disturbance or congenital vascular anomaly. It was defined based on history and clinical manifestations (e.g., large fetal head in proportion to pelvic outlet, prolonged labor, breech presentation, mechanical assistance with delivery), transfontanel cranial ultrasonography or CT results and knowledge of the birth weight-specific risks of the type of hemorrhage.

*Periventricular leukomalacia (PVL)* was defined as presence of focal necrotic lesions in the periventricular white matter and/or more diffuse white matter damage as indicated by results of cranial ultrasound and/or other imaging modalities, especially when performed on infants with intraventricular hemorrhage and/or ventriculomegaly who are at high risk for PVL.

*Pregnancy induced hypertension* (maternal hypertension) was defined as development of new arterial hypertension in the pregnant woman after 20-weeks gestation without presence of protein in the urine.

### Infant neurological international battery (INFANIB)-Chinese version

#### Translation and cultural adaptation

The procedures and criteria for assessment of the 20-item INFANIB scale were translated into Chinese by two doctors specializing in developmental pediatrics. The initial version was then translated back into English by an English teacher who was not knowledgeable about developmental pediatrics. The English version was sent to a professor in the Department of Physical Therapy of Hung Kuang University who had substantial experience in assessment using the INFANIB. The professor revised and approved this Chinese version of the INFANIB.

#### Assessment

All infants were assessed with the Chinese version of the INFANIB at months 3, 7 and 10 (corrected age for premature infants) by three medical staff members: a childcare physician (rater A), a nurse with 5 years experience in a neonatal ICU (rater B) and a physical therapist (rater C). The cut points for INFANIB scores were established as described previously [15]. For infants <4 months old, abnormal ≤ 48, transient = 49–65, and normal ≥ 66. For infants 4 to 8 months old, the cut points were abnormal ≤ 54, transient = 55–71, and normal ≥ 72. For infants ≥8 months old, the cut points were abnormal ≤ 68, transient = 69–82, and normal ≥ 83.

### Determination of gross motor development outcomes by PDMS

At age one year, all infants underwent assessment for motor development outcome by professional evaluators who used the Peabody Developmental Motor Scales, Second Edition (PDMS-2) for quantitative and qualitative evaluation of motor development [18]. The PDMS includes two independent scales to evaluate gross and fine motor development in children: the gross motor evaluation scale comprises 151 items to detect reflexes, equilibrium, acquirement and release, stationary and locomotion; the fine motor evaluation scales comprise 98 items to evaluate grasping, use of hands, visual-motor integration and object manipulation [9,18]. Reliability and validity of the PDMS-2 Chinese version was reported by Yang et al. in 2010 (in Chinese)[19]. In addition, experts in developmental pediatrics evaluated the infants for cerebral palsy (CP), basing their diagnosis on severe abnormal motor development outcomes and the international criteria for CP [20]. A Gross Motor Quotient (GMQ) score in the PDMS-2 of ≤79 was defined as motor development retardation [9]. Infants without CP and a normal PDMS-2 score were considered to have normal motor development. Parents accompanied their infants to promote optimal performance during assessment with the PDMS-2. Infants with normal neuromotor development at age one year were subsequently (12–24 months) followed up in the clinic using PDMS-2 evaluation and those with abnormalities received rehabilitation training in our hospital. Infants with transient stage were followed up once monthly. During the follow-up period, a physical therapist provided guidance and advice for rehabilitation training.

### Reliability and validity

#### Reliability

The interclass correlation coefficient (ICC-inter) and the intraclass correlation coefficient (ICC-intra) were used to assess reliability. Assessment of infants was repeated 3–5 days after the first assessment. The relationship of scores between rater B and C was used to determine the test-retest reliability. The relationship of scores among the three raters was used to measure intergroup reliability.

#### Validity

The PDMS-2 was used as the gold standard for measuring motor development. The presence of CP or GMQ ≤79 on the PDMS-2 was considered to be a motor development disorder. “Abnormal and transient” on INFANIB assessment was defined as positive for neuromotor developmental disorders, and “normal” on INFANIB assessment was defined as negative for neuromotor developmental disorders. When the results were transient, infants were followed up with repeat PDMS evaluations and at-home rehabilitation was recommended without help from professionals. The sensitivity, specificity, PPV and NPV were calculated accordingly.

### Statistical analysis

The clinical characteristics and INFANIB score are presented as mean ± standard deviations (SD) for continuous variables for preterm and full-term infants and compared using two-sample *t*-test. Categorical variables are presented as n (%) and compared using Pearson Chi-square test or Fisher’s exact test if the number of cells is less than five. The inter- and intraobserver differences of INFANIB measurements were evaluated by their reproducibility. An ICC > 0.90 shows high reliability, 0.75-0.90 reveals good reliability, 0.50-0.75 displays intermediate reliability and <0.05 suggests poor reliability. Sensitivity, specificity, PPV and NPV are shown for the predictive results of INFANIB assessments according to the PDMS evaluation. All statistical assessments were two-tailed and considered significant at *p* < 0.05. Statistical analyses were performed using SPSS 15.0 statistics software (SPSS Inc, Chicago, IL, USA).

## Results

Table 1 summarizes clinical characteristics for preterm and full-term infants. The preterm infants had significantly shorter gestational age and lower birth weight. A significantly higher proportion of full-term infants had asphyxia, brain damage and hyperbilirubinemia (Table 1). Ultrasound scans performed 24–72 h after birth for preterm infants showed local or extensive echo enhancement in tissues surrounding the cerebral ventricle while ultrasound scans for full-term infants showed spotty local or diffuse echo enhancement in tissues surrounding the cerebral ventricle accompanied by changes in cerebral ventricular shape and fuzzy structure and intracranial hemorrhage. The INFANIB and PDMS scores of all subjects were measured with questionnaires at 3, 7 and 10 months after birth. The ICC-inter values of interobserver differences for each age group all showed excellent assessments. INFANIB scores of two raters on two occasions (test-retest) were used to assess intraobserver reliability. The ICC-intra of the intraobserver differences of rater B shows that the assessments were excellent at 3 months and 7 months and were substantial at 10 months (0.68). The ICC-intra values for intraobserver differences of rater C were excellent for all three age groups. At 3, 7, and 10 months, no significant differences were observed in INFANIB scores or diagnostic results between preterm and full-term infants (Table 2). According to the PDMS evaluation and diagnosis criteria for cerebral palsy,13 preterm infants were diagnosed as having CP or movement retardation and 42 preterm infants were normal. Among the full-term infants, 13 infants were diagnosed as having CP or movement retardation and 36 were normal. Although abnormal PDMS results may indicate movement retardation or CP, not all children whose results indicate movement retardation also meet the criteria for CP. Table 3 shows the sensitivity, specificity, PPV and NPV of the INFANIB based on comparison with the observed results obtained using the PDMS for preterm infants. INFANIB had relatively high values in sensitivity, specificity, PPV and NPV at age of 10 months. However, the predictive results of preterm infants’ status from INFANIB at 3 months and 7 months were much lower values (Table 3).

**Table 1:** Clinical characteristics of preterm and full-term infants (N = 104)

**Variables**^**a**^	**Preterm infants (n = 55)**	**Full-term infants (n = 49)**	***p *-value**
GA, wk	33.18 ± 2.73 (Range: 27.29 to 36.86)	39.02 ± 1.13 (Range: 37.43 to 41.71)	<0.001^*^
BW, g	2260.78 ± 616.74 (Range:1040 to 3700)	3139.39 ± 390.43 (Range: 2330 to 3850)	<0.001^*^
Status at birth			<0.001^*^
Asphyxia	3 (5.5%)	22 (44.9%)	
Normal	52 (94.5%)	27 (55.1%)	
Complications			
Brain damage ^b^	10 (18.2%)	22 (44.9%)	0.003^*^
Intracranial hemorrhage	0 (0%)	7 (14.3%)	-
Maternal hypertension during pregnancy	10 (18.2%)	5 (10.2%)	0.248
Hyperbilirubinemia	15 (27.3%)	24 (49.0%)	0.022^*^
Neonatal mechanical ventilation over 48 h	4 (7.3%)	5 (10.2%)	0.732

^a ^Data are presented as mean ± SD with a range (minimum to maximum) for continuous variables and n (%) for categorical variables.

^b ^Brain damage includes hypoxic ischemic encephalopathy (HIE) of Full-term infants and PVL of Preterm infants.

* *p* < 0.05 indicates significant difference between preterm and full-term infants.

Abbreviations: GA, gestational age; BW, birth weight; HIE, hypoxic ischemic encephalopathy; PVL, periventricular leukomalacia.

**Table 2:** **Summary of INFANIB scores and diagnostic results for preterm and full-term infants**^**a**^

		**Preterm infants (n = 55)**	**Full-term infants (n = 49)**	***p*-value**
3 months				
	INFANIB score	59.60 ± 9.44	58.78 ± 8.72	0.514
	Diagnostic results			0.431
	Normal	27 (49.1%)	18 (36.7%)	
	Transient	15 (27.3%)	16 (32.7%)	
	Abnormal	13 (23.6%)	15 (30.6%)	
7 months				
	INFANIB score	64.82 ± 9.72	66.47 ± 9.92	0.313
	Diagnostic results			0.591
	Normal	26 (47.3%)	28 (57.1%)	
	Transient	13 (23.6%)	9 (18.4%)	
	Abnormal	16 (29.1%)	12 (24.5%)	
10 months				
	INFANIB score	78.56 ± 8.36	78.14 ± 9.64	0.687
	Diagnostic results			0.813
	Normal	36 (65.5%)	29 (59.2%)	
	Transient	8 (14.5%)	8 (16.3%)	
	Abnormal	11 (20.0%)	12 (24.5%)	

^a ^Data are shown as mean ± SD for INFANIB score with Mann–Whitney *U *test and n (%) for the diagnostic results with Pearson chi-square test.

**Table 3:** Summary of the predictive results of INFANIB for preterm infants (N = 55)

		**Observed outcomes from PDMS**					
**Months**	**Diagnostic from INFANIB**	**Abnormal**^**a **^**(n = 13)**	**Normal (n = 42)**	**Sensitivity**	**Specificity**	**PPV**	**NPV**
3							
	Abnormal^b ^(n = 28)	10	18	76.9%	57.1%	35.7%	88.9%
	Normal (n = 27)	3	24				
7							
	Abnormal^b ^(n = 29)	11	18	84.6%	57.1%	37.9%	92.3%
	Normal (n = 26)	2	24				
10							
	Abnormal^b ^(n = 19)	11	8	84.6%	81.0%	57.9%	94.4%
	Normal (n = 36)	2	34				

^a ^Abnormal in PDMS includes cerebral palsy and movement retardation (GMQ ≤79).

^b ^Abnormal category includes abnormal or transient motor development delay on INFANIB.

Abbreviations: PDMS, Peabody Developmental Motor Scales; PPV, positive predictive value; NPV, negative predictive value.

Table 4 shows the sensitivity, specificity, PPV and NPV of the INFANIB based on comparison with the observed results obtained by using the PDMS for full-term infants. The predictive values of full-term infants’ status from INFANIB were relatively high at ages of 7 and 10 months but, again, were much lower for specificity and PPV at 3 months (Table 4).

**Table 4:** Summary of the predictive results of INFANIB for full-term infants (N = 49)

		**Diagnostic results from PDMS**					
**Months**	**Diagnostic from INFANIB**	**Abnormal**^**a **^**(n = 13)**	**Normal (n = 36)**	**Sensitivity**	**Specificity**	**PPV**	**NPV**
3							
	Abnormal^b ^(n = 31)	10	21	76.9%	41.7%	32.3%	83.3%
	Normal (n = 18)	3	15				
7							
	Abnormal^b ^(n = 21)	11	10	84.6%	72.2%	52.4%	92.9%
	Normal (n = 28)	2	26				
10							
	Abnormal^b ^(n = 20)	12	8	92.3%	77.8%	60%	96.6%
	Normal (n = 29)	1	28				

^a ^Abnormal in PDMS includes cerebral palsy or movement retardation (GMQ ≤79).

^b ^Abnormal category includes abnormal or transient motor development delay on INFANIB.

Abbreviations: PDMS, Peabody Developmental Motor Scales; PPV, positive predictive value; NPV, negative predictive value.

## Discussions

In this study, assessment of a Chinese version of the INFANIB demonstrated that it had acceptable validity and reliability and was feasible for screening neuromotor developmental disorders in Chinese primary care settings. In China, the scales used most frequently in children's hospitals that offer comprehensive medicine and services, e.g. BSID-II, Gesell Developmental Schedules, PDMS-2, and AIMS [7-10], are time-consuming to administer and professional staff require specialized training at professional agencies. Therefore, these scales are impractical for wide application in Chinese primary care settings because of the heavy patient load, shortage of physicians, limited medical resources and low medical literacy in many community medical facilities. The most frequently applied scale for assessment of developmental milestones in China is the Denver Developmental Screening Test (DDST) [21]. However, the low sensitivity of this scale makes it unsuitable for identifying motor development retardation in the first year of life [21], which is likely due to the relatively small number of developmental milestones in the infancy stage. The INFANIB is a scale with favorable reliability and validity for the assessment of neuromotor development [14,22]. Results of our assessment of the feasibility of using the INFANIB in Chinese primary care settings showed that all intergroup ICC-inter values were higher than 0.8, demonstrating high reliability. The ICC-intra values of intraobserver (test-retest) reliability were also higher than 0.8 for rater C and for rater B at 3 months and 7 months. These results suggest that the INFANIB is a reliable and stable scale, and that medical staff with different medical backgrounds can master the application of this scale as long as the raters have knowledge of neuromotor development. In addition, administration of the INFANIB in this study was time-saving (mean time: 8 minutes), which is consistent with previous reports [14,22]. Given these findings, we believe that the INFANIB can be widely applied in Chinese primary care settings for screening motor development disorders in high-risk infants.

To measure validity, we used the PDMS-2 as the gold standard [8]. Studies have shown this scale to be a valid evaluative measure of infants receiving physical therapy, and infants with cerebral palsy and motors delays [18,23]. The PDMS-2 is widely accepted for the assessment of simple motor development; abnormal results in the PDMS may indicate movement retardation and cerebral palsy but it must be noted that all children identified to have movement retardation do not meet the defined criteria for cerebral palsy. In this study, the INFANIB had good sensitivity and negative predictive value for both preterm infants and full-term infants compared with a previous report [14]. However, for full-term infants at age 3 months, the specificity and positive predictive value were relatively low, suggesting that there were a relatively high number of false positives. The lower validity for 3-month-old infants can possibly be explained by results of the INFANIB development study in 1985 [15]. Children in that study were aged from 0 to 22 months and validity was observed to increase with age. The scale is therefore specifically recommended for use in evaluating infants aged 4–18 months of age [15]. In assessments using the INFANIB, infants are assessed in the supine, prone, sitting, standing and suspended positions for body tone, posture, French angles and primitive reflexes to screen them for abnormal motor development. These items involve the tension of trunk extensors, lower limb extensors and adductors, which may not be easy to measure at the early stage of infancy. In addition, since the early stage of infants might have better brain development plasticity, INFANIB results assessment may become unclear after the infants receive rehabilitation. The most likely explanation, however, is that poor predictive power is related to poor prediction of the single items themselves at this early age.

In preterm infants at age 3 and 7 months (corrected age), the specificity and positive predictive value were relatively low compared with that previously reported [14]. Pedersen et al. (2000) also reported that the validity of the INFANIB for predicting the future presence of CP was low in premature infants weighing < 2000 g (birth weight) and aged 7 months (corrected age) since only 8 of 65 infants who were dystonic at 7 months developed suspected CP [23]. Evidence shows transient dystonia to different extent in premature infants aged 4–8 months. Several studies have confirmed that the gross motor development in premature infants (including healthy premature infants) is significantly different from that in full-term infants, and is characterized by abnormal posture, abnormal muscular tone and asymmetric position, at 4–8 months corrected age, which resolves at the age of 8–12 months [24-28]. This may explain why the validity of INFANIB for predicting the presence of CP at the age of 7 months was low in premature infants. It should be noted that three preterm infants in this study were diagnosed as having asphyxia based primarily on Apgar scores (0–3 within 5 minutes of birth is defined as asphyxia) [29], and supplemented with abnormal pH of umbilical artery blood (<7.0) and the presence of neurological symptoms (e.g., convulsions, coma, hypotonia) and symptoms of cardiovascular, gastrointestinal, blood, respiratory and/or urinary system indications as criteria for neonatal asphyxia.

## Conclusions

In conclusion, assessment using the INFANIB is simple and time-saving, and the scale has favorable reliability and acceptable validity at the age of 10 months for both premature and full-term infants at high-risk for developmental disorders. The INFANIB is therefore an appropriate tool for screening neuromotor developmental disorders in Chinese primary care settings. For premature infants at the age of 7 months or less, and for full-term infants at the age of 3 months or less, detecting abnormalities using the INFANIB does not lead to the conclusion of motor development delay, even in high-risk infants.

### Limitations

Although our results are useful for follow-up of preterm and term neonates at risk of neurologial problems in a Chinese population, the relatively small number of infants studied and the lack of a control group limits the strength of study conclusions. We chose not to use a control group in this study because, while training the three raters and gaining early experience with INFANIB in full-term infants and infants with low risk in our Health Care Clinic, our results showed that scores were in the normal cut-off point as referenced in the previously published U.S. study by Ellison, the developer of the scale [15]. Since the present study aimed to investigate a Chinese version of INFANIB that would be used with Chinese children, of course controls would have been useful. Additional multicenter study will be conducted with a large sample and longer neurological outcome to establish cut-off criteria for evaluation with INFANIB in Chinese populations. Also, technical, linguistic, and conceptual problems involved in the translation of questionnaires [30,31], limited the ability to compare results of studies using the same instruments in a different language. We also must note that the poor predictive power seen in infants aged 3 months may be related to the poor prediction of the single items at this age, suggesting that only one neurological examination is an insufficient basis for prognosis, especially at early ages.

## Abbreviations

CP, Cerebral palsy; GMQ, Gross motor quotient; HIE, Hypoxic ischemic encephalopathy; INFANIB, Infant Neurological International Battery; NICU, Neonatal intensive care unit; PDMS, Peabody Developmental Motor Scales; PVL, Periventricular leukomalacia.

## Competing interests

The authors declare that they have no competing interests.

## Authors’ contributions

CZ designed the study and wrote the protocol. WL participated in study design and coordination and helped to draft the manuscript. QC and CL collected data during the study period and follow-up. ZH managed the literature searches and analyses. KL provided statistical analysis. EW wrote the first draft of the manuscript. All authors read and approved the final manuscript.

## Pre-publication history

The pre-publication history for this paper can be accessed here:

http://www.biomedcentral.com/1471-2431/12/72/prepub
